# Sequential Treatment with Regorafenib and Trifluridine/Tipiracil ± Bevacizumab in Refractory Metastatic Colorectal Cancer in Community Clinical Practice in the USA

**DOI:** 10.3390/cancers17060969

**Published:** 2025-03-13

**Authors:** Daniel H. Ahn, Tanios S. Bekaii-Saab, Chengbo Yuan, Milena Kurtinecz, Xiaoyun Pan, Zdravko Vassilev, Federica Pisa, Helene Ostojic

**Affiliations:** 1 Mayo Clinic, Phoenix, AZ 85054, USA; bekaii-saab.tanios@mayo.edu; 2 Real World Evidence Oncology, Bayer HealthCare Pharmaceuticals, Whippany, NJ 07981, USA; 3 Bayer Consumer Care AG, 4052 Basel, Switzerland; 4 Real World Evidence Oncology, Bayer AG, 13342 Berlin, Germany

**Keywords:** Regorafenib, Trifluridine/Tipiracil, TAS-102, colorectal cancer, refractory, metastatic, community practice, sequence, real world

## Abstract

**Background**: Regorafenib (R) and Trifluridine/Tipiracil ± bevacizumab (T) are approved for treating refractory metastatic colorectal cancer (mCRC) but their optimal sequence is unclear. This study describes the characteristics/clinical outcomes of patients with mCRC in U.S. clinical practice treated sequentially with R-T or T-R. **Methods**: A retrospective cohort study of 818 patients treated with R-T or T-R between January 2015 and November 2022 was conducted using an electronic health record-derived database. The primary objective was to describe the demographic/clinical characteristics and biomarker status of patients treated with R-T or T-R, stratified by treatment line/age. Secondary objectives were to evaluate/estimate the frequency of neutropenia and myelosuppression-related treatments, the number/type of subsequent therapies, time to treatment discontinuation (TTD), and overall survival (OS). **Results**: Baseline characteristics were similar among patients who received R-T (n = 393) or T-R (n = 425). Lower rates of moderate/severe neutropenia (26%/12% vs. 32%/16%) and granulocyte colony-stimulating factor/erythropoietin use (22% vs. 24%) were observed with R-T versus T-R. The median TTD was 8.7 months and 8.5 months with R-T versus 8.1 months and 7.9 months with T-R as third- and fourth-line treatment, respectively. The median OS was 13.1 months and 11.6 months with R-T versus 11.5 months and 10.3 months with T-R as third- and fourth-line treatment, respectively. **Conclusions**: This study did not show a statistically significant difference in OS with R-T versus T-R. Although limited by its retrospective nature, the study suggested R-T may be preferable to T-R given the observed reduction in neutropenia/myelosuppression-related treatments.

## Simple Summary

Regorafenib and Trifluridine/Tipiracil with or without bevacizumab are approved treatments for refractory metastatic colorectal cancer (mCRC). However, the preferred sequence of these treatments is not clear. This study aimed to determine whether treatment with Regorafenib before or after Trifluridine/Tipiracil (with or without bevacizumab) was the most effective treatment sequence in patients with mCRC in real-world clinical practice (i.e., not as part of a clinical trial) in the USA. A statistically significant difference in the time patients lived following treatment with Regorafenib followed by Trifluridine/Tipiracil compared with the reverse sequence was not determined, but patients who received Regorafenib first appeared to have lower rates of decreased bone marrow activity (myelosuppression), decreased white blood cell counts (neutropenia), and related medical interventions for these conditions. Overall, these findings suggest that treatment with Regorafenib before Trifluridine/Tipiracil may be preferable; however, patients should be assessed on an individual basis to determine the most suitable approach.

## 1. Introduction

Colorectal cancer (CRC) is the fourth most frequently diagnosed cancer in the USA, and there were an estimated 152,810 new cases (7.6% of all new cases of any type of cancer) and an estimated 53,010 deaths (8.7% of all cancer-related deaths) in 2024 [1]. The 5-year relative survival rate of patients with CRC in the USA is 91% when localized and 74% when regional, but only 16% when metastatic to distant sites (other than lymph nodes) [1]. Moreover, since around 20% of newly diagnosed patients with CRC will have metastatic disease and another 25% who present with early-stage disease later develop metastases [2], almost half of all patients will likely require multiple lines of therapy.

Current standard-of-care therapies for metastatic CRC (mCRC) include fluoropyrimidine-based chemotherapy and anti-vascular endothelial growth factor (VEGF) or anti-epidermal growth factor receptor (EGFR) treatments for patients with *KRAS*/*NRAS* wild-type disease [3]. Immunotherapies are available for patients with microsatellite instability-high or deficient mismatch repair disease [3]. Regorafenib (R), a multikinase inhibitor active against several angiogenic and other oncogenic receptor tyrosine kinases, and Trifluridine/Tipiracil (TAS-102), a cytotoxic combination of a thymidine-based nucleic acid analog and a thymidine phosphorylase inhibitor, are treatment options for refractory mCRC with distinctly different safety profiles [4,5,6,7]. Both R and TAS-102 ± bevacizumab (T) are approved by the U.S. Food and Drug Administration (FDA) and European Medicines Agency (EMA) for the treatment of refractory mCRC based on the proven efficacy of both drugs in phase 3 trials [5,6,8,9,10,11,12]. The collection and evaluation of data on the sequencing of treatments in patients with refractory mCRC is important for providing information that may help in appropriate treatment selection for individual patients, thereby enabling patients to receive all approved therapies that may lead to improved outcomes [13]. Moreover, antiangiogenic drugs are potential chemosensitizers for chemotherapy [14]; therefore, real-world data on the order of use of antiangiogenic agents such as R and chemotherapeutic agents such as TAS-102 may provide further insights into optimal drug sequencing. Since R and TAS-102 have different mechanisms of action and safety profiles, patient performance status, comorbidities, and potential adverse effects are key factors for consideration when sequencing these drugs, particularly for patients who may have already received multiple lines of cytotoxic chemotherapy. These patients may, therefore, have compromised bone marrow reserves and may have experienced, and potentially still show signs of, associated toxicities [4,15,16]. The optimal sequence of R and T has not been evaluated in a randomized prospective trial, and sequencing data from prior observational studies and a meta-analysis of retrospective observational comparisons of the two drugs have provided mixed results that have not established the optimal order of their use [15,16,17,18,19,20,21,22,23]. Our real-world study provides an up-to-date analysis that takes into account any changes to clinical practice in the USA since prior studies were conducted, resulting from recent label changes to either study drug, that may have affected clinical benefit or safety.

The aim of this real-world study was to describe the demographic and clinical characteristics and clinical outcomes of patients with mCRC treated sequentially with R followed by T (R-T) or T followed by R (T-R) in community oncology practice in the USA. Specifically, the primary objective was to describe the demographic and clinical characteristics, and biomarker status, of patients with mCRC who received sequential treatment with R-T or T-R, stratified by line of treatment and by age group (<65 and ≥65 years). Secondary objectives were to describe the subsequent therapies and number of subsequent lines of treatment after sequential treatment, the frequency and incidence of neutropenia and myelosuppression-related medical interventions during sequential treatment, the duration of sequential therapy, and to estimate overall survival (OS) of patients treated with R-T or T-R in third- and fourth-line settings, stratified by age group, line of treatment, prior bevacizumab, and *KRAS* mutation status.

## 2. Methods

This was a retrospective observational cohort study that used the nationwide Flatiron Health (New York, NY, USA) longitudinal electronic health record (EHR)-derived deidentified database. The database comprises deidentified patient-level structured and unstructured data, curated via technology-enabled abstraction [24,25]. During the study period, the deidentified data were collected from approximately 280 U.S. cancer clinics (mostly community oncology practices) and include over 35,000 adult patients who had a diagnosis of mCRC. The deidentified data are obligated to prevent reidentification and protect confidentiality.

**Figure 1:**
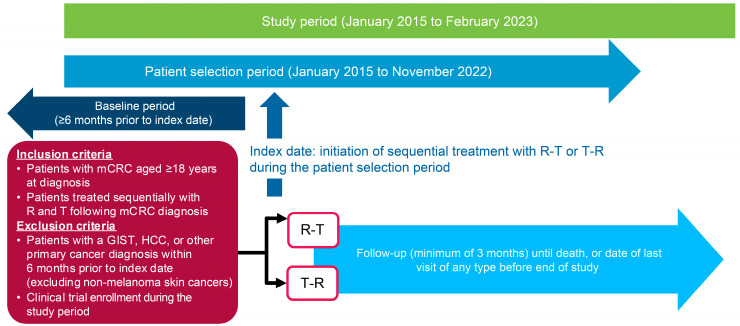
Study schema. GIST: gastrointestinal stromal tumor, HCC: hepatocellular carcinoma, mCRC: metastatic colorectal cancer, R: Regorafenib, T: TAS-102 ± bevacizumab.

A broad cohort of patients with CRC in the overall cancer database was further subject to selection criteria to generate an analysis cohort of eligible cases with mCRC. Eligible patients were selected from the database who had been diagnosed with mCRC, were aged ≥18 years at mCRC diagnosis, and were initiated on sequential treatment with R-T or T-R between 1 January 2015, and 30 November 2022, with no limit on the time interval between finishing the first treatment and starting the second treatment in the sequence, but without any intervening line of therapy (Figure 1). The index date was defined as the start date of the first therapy in the sequence, and patients were divided into two cohorts (R-T or T-R) based on their index sequence. Patients were followed for a minimum of 3 months from the index date to the earliest of the following events: date of death, the date of last evidence of patient’s activity in the database (i.e., last visit of any kind), or the end date of the study period (1 January 2015–28 February 2023). Exclusion criteria included a diagnosis of gastrointestinal stromal tumor, hepatocellular carcinoma, or another primary cancer (except non-melanoma skin cancer) in the baseline period (≥6 months prior to the index date) or involvement in a clinical trial during the study period.

The primary endpoints were demographic and clinical characteristics, and biomarker status (*KRAS* and *BRAF* mutation), of the R-T and T-R cohorts. The study also evaluated several secondary endpoints, including the proportion of patients receiving subsequent therapy after the R-T or T-R index sequence, number of subsequent lines of treatment, and type of agent (i.e., chemotherapy, targeted medicine, immunotherapy, other) received. The incidence of neutropenia (classified as mild, >1.0–1.5 × 10^9^ neutrophils/L; moderate, 0.5–1.0 × 10^9^ neutrophils/L; severe, <0.5 × 10^9^ neutrophils/L) was measured during the period of sequential index treatment with R-T or T-R. The use of myelosuppression-related medical intervention was assessed based on primary prophylactic granulocyte colony-stimulating factor (G-CSF) initiated during Days 0–13, with Day 0 defined as the start of index treatment, secondary prophylactic G-CSF initiated during Days 14–18, and overall use of G-CSF initiated from Day 0 to the end of study (index) treatment. Further secondary endpoints were duration of the index sequential therapy and OS. Duration of index therapy was defined by time to treatment discontinuation [TTD] (i.e., the period between the start and end dates of the line index sequence). Patients were discontinued at the end of sequential therapy or death date, or earlier if the patient (1) advanced to another line of therapy, or (2) did not advance to another line of therapy and did not have a visit at least 120 days after the end of sequential treatment; patients who did not meet any of these criteria were censored at the end of sequential therapy. OS was calculated from the index date to the date of death due to any cause; patients alive at the end of follow-up or lost to follow-up were censored at the last date known to be alive (i.e., the earliest of last day of follow-up, last date of activity, or end of study).

### 2.1. Statistical Analysis

This retrospective observational study was descriptive in nature and took an explorative approach; therefore, there was no hypothesis testing, and the study was not designed to test statistical significance. Descriptive analysis for all baseline and clinical characteristics were performed by cohort for all patients who initiated their index sequence in any line of treatment. Frequencies and percentages were calculated for categorical variables and mean/standard deviation and median/range for continuous variables. Incidence rates of myelosuppression intervention for the entire cohort of patients, defined as the number of new cases during the index R-T or T-R sequential therapeutic period, were calculated as the number of events divided per person-time and expressed as events per 1000 person-months.

The time-to-event outcomes OS and TTD were analyzed in patients receiving sequential therapy of R-T and T-R, respectively, in third- and fourth-line settings. Median OS and TTD were obtained using the Kaplan–Meier product limit estimator. Cox proportional hazards regression models were used to estimate crude hazard ratios (HRs) and adjusted HRs, with 95% confidence intervals (95% CIs). The proportional hazards assumption was tested with a supremum test. Variables in both adjusted models included age, gender, Eastern Cooperative Oncology Group performance status (ECOG PS), *KRAS* mutation status, prior targeted treatments (anti-EGFR or bevacizumab), stage at initial diagnosis, tumor sidedness, and site of metastasis.

For the subgroup analyses, Cox proportional hazards models were applied to provide unadjusted and adjusted HRs including covariates of index line, age, gender, ECOG PS, *KRAS* mutation status, prior anti-EGFR therapy, prior bevacizumab, stage at initial diagnosis, tumor sidedness, and site of metastasis. HRs were provided for OS and TTD (R-T vs. T-R cohorts) by age, line of therapy, prior bevacizumab, *KRAS* mutation status, site of metastasis, and tumor sidedness among third- and fourth-line patients combined.

As this was a retrospective observational study, the effective sample sizes were based on the total number of patients with non-missing data for the parameter of interest.

### 2.2. Ethics Approval and Consent to Participate

This analysis was a secondary data analysis based on patient healthcare information from a deidentified database, from which patients could not be reidentified. As dictated by Title 45 Code of Federal Regulations (45 CFR 46.101(b)(4)) (available at https://www.govinfo.gov/content/pkg/CFR-2011-title45-vol1/pdf/CFR-2011-title45-vol1.pdf (accessed on 7 March 2025)), this analysis was conducted under an exemption from Institutional Review Board oversight for U.S.-based studies using deidentified healthcare records, and for which informed consent was not applicable.

## 3. Results

### 3.1. Patients

During the study period, 963 patients received sequential treatment with R-T or T-R, and of these, 818 patients (n = 393 R-T; n = 425 T-R) met all study inclusion criteria (Supplementary Figure S1). Patients had a median age of 63 years, 56% were male, 56% had Stage IV disease at diagnosis, and 71% had ECOG PS 0 or 1. Most patients received index therapy (first drug in the sequence R-T or T-R) as their third line (42%) or fourth line (24%) of treatment; other patients (34%) received earlier or later lines of treatment. Baseline characteristics were similar between cohorts (Table 1). The proportion of patients who received TAS-102 in combination with bevacizumab were similar in both groups regardless of the index line of treatment (45/393 patients [11%] in the R-T group and 63/425 patients [15%] in the T-R group). Baseline characteristics were also similar between cohorts when stratified by age and in patients receiving sequential treatment as third- and fourth-line therapy (Supplementary Tables S1 and S2).

### 3.2. Neutropenia and Myelosuppression

Moderate or severe neutropenia was less frequently reported with R-T versus T-R during the therapeutic period (26% vs. 32% and 12% vs. 16%, respectively; Table 2). The frequency of G-CSF or erythropoietin taken during the sequential treatment period and overall was also lower with R-T versus T-R (22% vs. 24% and 14% vs. 18%, respectively; Table 2) and the incidence rate of myelosuppression intervention was lower for R-T versus T-R (14.9 vs. 22.2 per 1000 person-months, respectively; Table 2).

### 3.3. Subsequent Systemic Cancer Therapies

Approximately one-third of the patients in each cohort received subsequent therapy, and the majority (70%) received only one additional line (median 1, range 1–5; Table 3). The frequency of chemotherapy-based subsequent therapy (including a combination with bevacizumab or targeted therapies) was higher following T-R (chemotherapy 40%, chemotherapy + bevacizumab 36%, and chemotherapy + targeted therapy 23%; Table 3) than following R-T (32%, 26%, and 20%; Table 3), while the frequency of subsequent targeted therapy was higher following R-T (24%; Table 3) than following T-R (10%; Table 3). The use of subsequent immunotherapy was less frequent than chemotherapy ± targeted therapy and similar in both cohorts (17% and 16%, following R-T and T-R, respectively; Table 3).

### 3.4. OS and Time to Discontinuation

**Figure 2:**
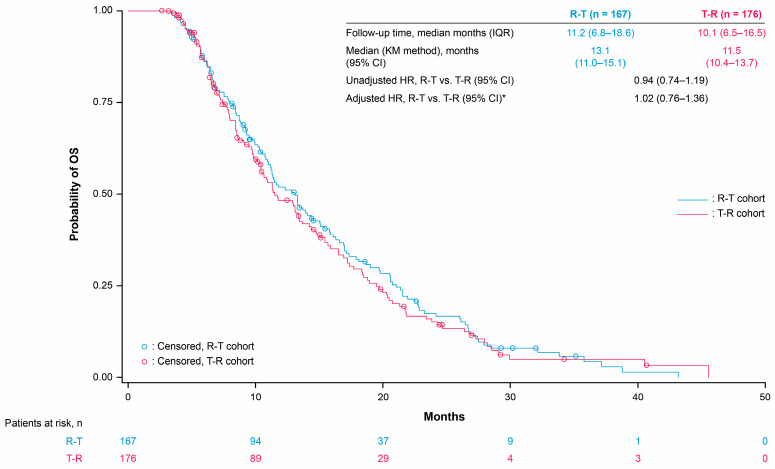
OS for R-T and T-R in third-line patients. * Adjusted for age, gender, ECOG PS, *KRAS* mutation status, prior targeted treatments (anti-EGFR or bevacizumab), stage at initial diagnosis, tumor sidedness, and site of metastasis. HRs (Cox proportional hazards model) are for exploratory purposes only (baseline not balanced). OS was calculated from the index date to the date of death due to any cause; patients who were alive at the data cut-off date were censored at the last confirmed activity date. CI: confidence interval, ECOG PS: Eastern Cooperative Oncology Group performance status, EGFR: epidermal growth factor receptor, HR: hazard ratio, IQR: interquartile range, KM: Kaplan–Meier, OS: overall survival, R: Regorafenib, T: TAS-102 ± bevacizumab.

**Figure 3:**
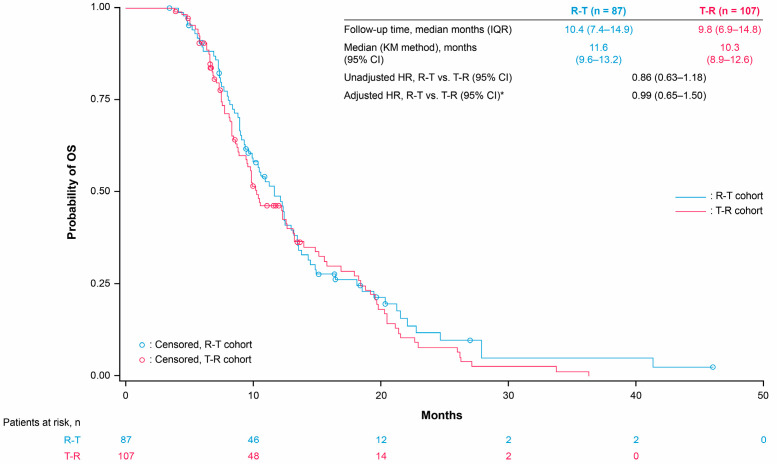
OS for R-T and T-R in fourth-line patients. * Adjusted for age, gender, ECOG PS, *KRAS* mutation status, prior targeted treatments (anti-EGFR or bevacizumab), stage at initial diagnosis, tumor sidedness, and site of metastasis. HRs (Cox proportional hazards model) are for exploratory purposes only (baseline not balanced). OS was calculated from the index date to the date of death due to any cause; patients who were alive at the data cut-off date were censored at the last confirmed activity date. CI: confidence interval, ECOG PS: Eastern Cooperative Oncology Group performance status, EGFR: epidermal growth factor receptor, HR: hazard ratio, IQR: interquartile range, KM: Kaplan–Meier, OS: overall survival, R: Regorafenib, T: TAS-102 ± bevacizumab.

**Figure 4:**
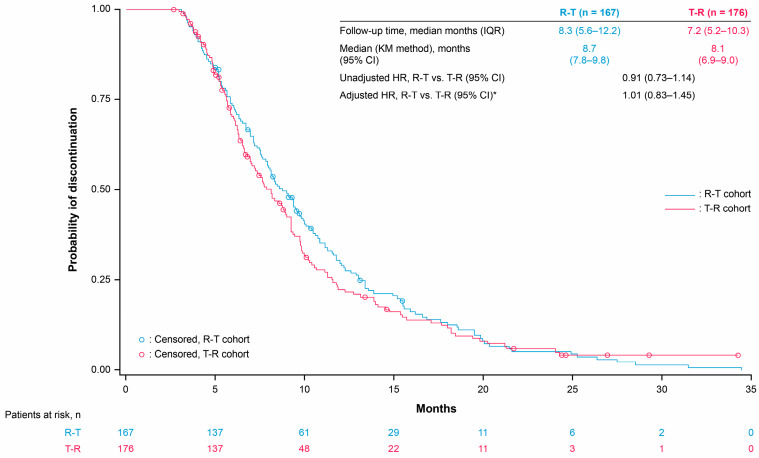
Time to discontinuation for R-T and T-R in third-line patients. * Adjusted for age, gender, ECOG PS, *KRAS* mutation status, prior targeted treatments (anti-EGFR or bevacizumab), stage at initial diagnosis, tumor sidedness, and site of metastasis. HRs (Cox proportional hazards model) are for exploratory purposes only (baseline not balanced). CI: confidence interval, ECOG PS: Eastern Cooperative Oncology Group performance status, EGFR: epidermal growth factor receptor, HR: hazard ratio, IQR: interquartile range, KM: Kaplan–Meier, R: Regorafenib, T: TAS-102 ± bevacizumab.

**Figure 5:**
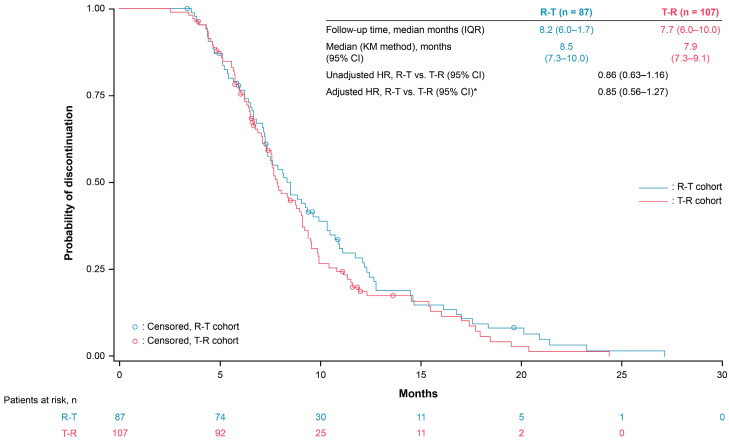
Time to discontinuation for R-T and T-R in fourth-line patients. * Adjusted for age, gender, ECOG PS, *KRAS* mutation status, prior targeted treatments (anti-EGFR or bevacizumab), stage at initial diagnosis, tumor sidedness, and site of metastasis. HRs (Cox proportional hazards model) are for exploratory purposes only (baseline not balanced). CI: confidence interval, ECOG PS: Eastern Cooperative Oncology Group performance status, EGFR: epidermal growth factor receptor, HR: hazard ratio, IQR: interquartile range, KM: Kaplan–Meier, R: Regorafenib, T: TAS-102 ± bevacizumab.

The median OS was 13.1 months and 11.6 months with R-T versus 11.5 months and 10.3 months with T-R in the third and fourth line, respectively (Figure 2 and Figure 3). The third- and fourth-line Cox proportional hazards model-adjusted HRs (R-T vs. T-R) were 1.02 (95% CI 0.76–1.36) and 0.99 (95% CI 0.65–1.50), respectively (Supplementary Table S3). Median TTD was 8.7 months and 8.5 months with R-T versus 8.1 months and 7.9 months with T-R in the third and fourth line, respectively (Figure 4 and Figure 5). The third- and fourth-line Cox proportional hazards model-adjusted HRs (R-T vs. T-R) were 1.01 (95% CI 0.83–1.45) and 0.85 (95% CI 0.56–1.27), respectively (Supplementary Table S4). The study was not designed to detect statistical significance.

### 3.5. Subgroup Analyses

**Figure 6:**
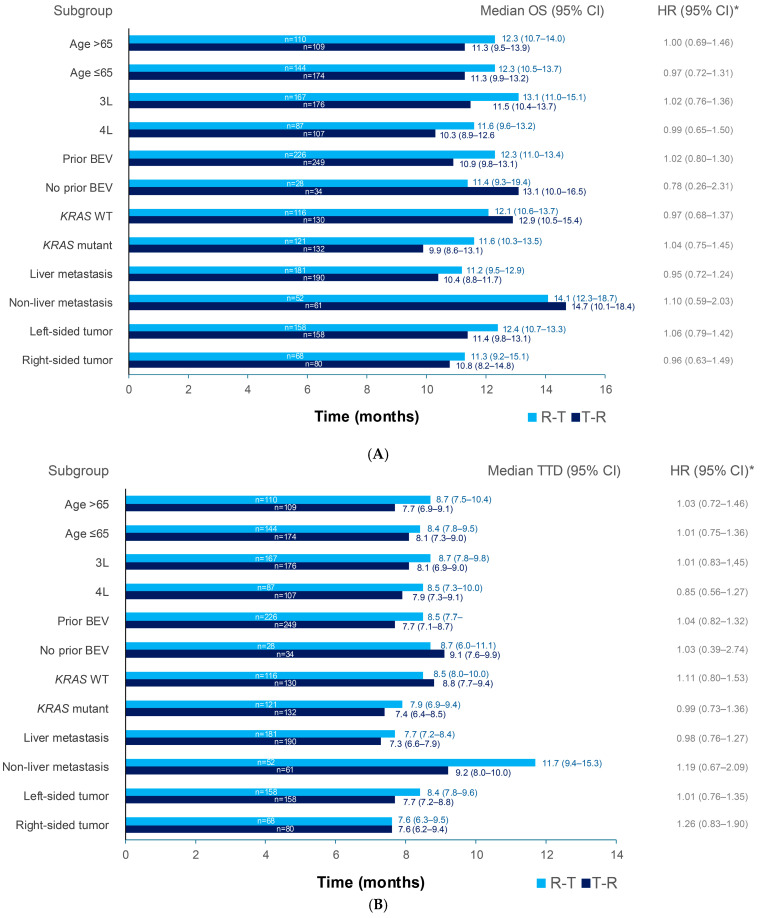
Median OS (**A**) and time to discontinuation (**B**) (months [95% CI]) across subgroups of patients treated with R-T or T-R. * HR adjusted for index line, age, gender, ECOG PS, *KRAS* mutation status, prior anti-EGFR, prior bevacizumab, stage at initial diagnosis, tumor sidedness, and site of metastasis. 3L: third line, 4L: fourth line, BEV: bevacizumab, CI: confidence interval; ECOG PS: Eastern Cooperative Oncology Group performance status, EGFR: epidermal growth factor receptor, HR: hazard ratio, OS: overall survival, R: Regorafenib, T: TAS-102 ± bevacizumab, TTD: time to treatment discontinuation, WT: wild type.

In subgroup analyses of OS, sequential treatment with R-T among third- and fourth-line patients appeared to show a trend towards longer median OS than T-R regardless of age category (≤65 or >65 years) and in patients with *KRAS* mutation or who had received bevacizumab in any prior line of therapy (Figure 6A and Supplementary Table S3). Analysis of OS by site of metastasis and tumor sidedness revealed that sequential treatment with R-T among third- and fourth-line patients appeared to show a trend towards longer OS than T-R in patients with liver metastases and in patients with either right-sided or left-sided tumors; T-R appeared to provide a minimal advantage in patients with non-liver metastases (Figure 6A and Supplementary Table S3).

In a subgroup analysis of TTD, sequential treatment with R-T among third- and fourth-line patients appeared to show a trend towards longer median TTD than T-R regardless of age category (≤65 or >65 years) and longer median TTD in patients with *KRAS* mutation or who had received bevacizumab in any prior line of therapy (Figure 6B and Supplementary Table S4). Analysis by site of metastasis and tumor sidedness showed that sequential treatment with R-T among third- and fourth-line patients appeared to show a trend towards longer TTD than T-R in patients with non-liver metastases and in patients with left-sided tumors; a trend towards a minimal advantage was seen for R-T in patients with liver metastases (Figure 6B and Supplementary Table S4).

## 4. Discussion

Although both Regorafenib and TAS-102 are approved therapies for third-line treatment of patients with mCRC (or second line following triplet chemotherapy) [8,9,10,11], the optimal sequence of their use remains unclear. One large (n = 866) Italian study showed that in the real-world setting, patients treated with Regorafenib first had longer OS and progression-free survival, and fewer hematologic adverse events than those who started with TAS-102 [15]. Several other prior real-world studies have provided inconsistent data regarding the sequential use of these two therapies [15,16,17,18,19,20,21,22,23]. Although most studies found no differences in survival benefit, some studies reported advantages of one sequence over another, either in favor of R-T or in favor of T-R. Moreover, several of these studies have been limited by small or unbalanced sample sizes and/or unbalanced or monoracial patient characteristics [17,18,20,21,22,23], and there remains a need for further and larger studies. In addition, more recent real-world data may take into account any effect on OS that may be due to the increased use of combined TAS-102 and bevacizumab, which was FDA- and EMA-approved in 2023 [9,10]. This approval was based on data from the phase 3 SUNLIGHT trial, in which the median OS was 10.8 months for this combination compared with 7.5 months for TAS-102 alone (HR 0.61, *p* < 0.001) [12].

Our real-world study suggested that in patients treated with sequential R-T, there was a modest trend towards longer OS and TTD when compared with T-R that may have clinical relevance; however, the study was descriptive in nature and not designed to detect statistically significant differences. The findings of this study were consistent with several other studies that have not shown a clinically and/or statistically significant difference in OS for R-T versus T-R [15,18,19,20,21,22,23]. In our study, a similar proportion of patients in both groups had received prior bevacizumab (78–79%). A similar proportion of patients had received bevacizumab in combination with TAS-102 as part of their sequential treatment (11–15%); in view of this limited sample size, further studies will address this patient population. A study in Asian patients suggested that exposure to prior targeted treatments such as bevacizumab may influence the length of survival benefit associated with Regorafenib treatment [26]. Since bevacizumab was used in combination with TAS-102 in some patients who received T-R in this study, increased exposure to prior bevacizumab in these patients compared with those who had received TAS-102 alone may have influenced the effectiveness of subsequent Regorafenib. The subgroup analysis suggested a trend towards a survival advantage for R-T versus T-R in patients who received prior bevacizumab, but the potential clinical significance of this observation was not evaluated due to the descriptive nature of the study.

Our study showed that there is a lower risk of neutropenia and reduced use of G-CSF with R-T compared to the reverse sequence of T-R. These findings suggest that the safety profile of the sequence may be dictated by the first drug used, leading to higher rates of hematologic adverse events and related interventions for T-R compared with R-T. These safety results are supported by other studies, including a systematic review and network meta-analysis of randomized controlled clinical trials showing that when compared with TAS-102, Regorafenib is associated with higher rates of grade 3 hand–foot skin reaction, fatigue, and hypertension, while TAS-102 is associated with a higher incidence of hematologic toxicities such as neutropenia [27].

Around a third of patients went on to receive further treatment (1–5 lines) in each group, suggesting different sequences may not impair the opportunity to receive subsequent therapies. The availability and use of improved third- and later-line chemotherapeutic options that help to maximize the number of therapies received may be regarded as one of the key drivers of improved OS of patients with mCRC over the last two decades [13,28]. In our study, among patients who went on to receive further treatment, the proportion of patients who received chemotherapy-based subsequent therapy was higher following T-R than following R-T, while the proportion of patients who received subsequent targeted therapy was higher following R-T than following T-R. These data may reflect the toxicities experienced related to the last therapy in the sequence, with further chemotherapy more likely to be used following Regorafenib.

The strength of this study is that it shows the characteristics and clinical outcomes of patients with mCRC treated in real-world clinical practice using a large EHR-derived database from community clinics in the USA. However, it shares limitations common to retrospective observational studies using real-world data, such as residual confounding due to factors unmeasured in the data source, high proportions of unknown or missing data for some variables (e.g., for neutropenia and some biomarkers), and unbalanced groups due to lack of randomization. A further limitation of the study is a lack of data on drug dose intensity and in particular any differences in the use of standard or flexible dosing strategies for Regorafenib, which have been evaluated in several recent studies (e.g., the phase 2 ReDOS study) [29,30,31,32], and which may have impacted the results. In addition, in our study there was a relatively small proportion of patients who received TAS-102 in combination with bevacizumab. Finally, a limitation of studies involving sequential treatments in the real-world setting is the introduction of a general survival benefit in both cohorts; although unbiased, this survival benefit should be considered when interpreting the results. Further randomized controlled studies are needed to evaluate more fully the sequencing of these two drugs in patients who receive TAS-102 in combination with bevacizumab.

## 5. Conclusions

This real-world retrospective large-scale U.S. study did not show any significant difference in OS with R-T versus T-R when treating patients with refractory mCRC in the third- and fourth-line setting. However, R-T may be preferable over the reverse sequence given the observed reductions in neutropenia, myelosuppression, and related medical interventions. As the data do not show a statistically significant difference in OS for R-T versus T-R, the optimal sequence strategy should be determined on a patient-by-patient basis.

## Figures and Tables

**Table 1:** Patient characteristics at baseline.

	R-T n = 393	T-R n = 425	Overall n = 818
**Median age (IQR), years**	63 (56, 70)	63 (56, 72)	63 (56, 71)
Age ≤ 65, n (%)	225 (57)	246 (58)	471 (58)
Age >65, n (%)	168 (43)	179 (42)	347 (42)
**Gender, n (%)**			
Male	233 (59)	227 (53)	460 (56)
Female	160 (41)	198 (47)	358 (44)
**Race, n (%)**			
White	241 (61)	270 (64)	511 (62)
Black or African American	64 (16)	54 (13)	118 (14)
Asian	19 (5)	16 (4)	35 (4)
Other	44 (11)	55 (13)	99 (12)
Unknown/missing	25 (6)	30 (7)	55 (7)
**Side of primary tumor, n (%)**			
Left	240 (61)	241 (57)	481 (59)
Right	112 (28)	115 (27)	227 (28)
Unknown/missing	41 (10)	69 (16)	110 (13)
**Site of metastasis, n (%)**			
Liver ± other metastatic sites	270 (69)	282 (66)	552 (67)
Non-liver only	94 (24)	91 (21)	185 (23)
Unknown/missing	29 (7)	52 (12)	81 (10)
**Stage at initial diagnosis, n (%)**			
0/I	13 (3)	12 (3)	25 (3)
II	39 (10)	50 (12)	89 (11)
III	116 (30)	119 (28)	235 (29)
IV	217 (55)	237 (56)	454 (56)
Unknown/missing	8 (2)	7 (2)	15 (2)
**ECOG PS, n (%)**			
0/1	278 (71)	300 (71)	578 (71)
2/3	31 (8)	43 (10)	74 (9)
Unknown/missing *	84 (21)	82 (19)	166 (20)
***KRAS* mutation, n (%)**			
Positive	183 (47)	194 (46)	377 (46)
Negative	167 (42)	192 (45)	359 (44)
Failed/indeterminate test	4 (1)	5 (1)	9 (1)
Unknown/missing	39 (10)	34 (8)	73 (9)
***BRAF* mutation, n (%)**			
Positive	5 (1)	19 (4)	24 (3)
Negative	228 (58)	268 (63)	496 (61)
Failed/indeterminate	7 (2)	4 (1)	11 (1)
Unknown/missing	153 (39)	134 (32)	287 (35)
**Line of index treatment, n (%)**			
1	26 (7)	27 (6)	53 (6)
2	67 (17)	67 (16)	134 (16)
3	167 (42)	176 (41)	343 (42)
4	87 (22)	107 (25)	194 (24)
5–8	46 (12)	48 (11)	94 (11)
**Time between metastatic diagnosis and index date, median months (IQR)**	24.6 (16.6, 35.7)	24.5 (17.3, 36.0)	24.6 (17.0, 35.9)
**Prior anti-EGFR, n (%) ^†^**	131 (33)	153 (36)	284 (35)
**Prior bevacizumab, n (%) ^†^**	308 (78)	337 (79)	645 (79)
**Neutropenia, n (%) ^‡^**			
Moderate (0.5–1 × 10^9^ neutrophils/L)	14 (4)	8 (2)	22 (3)
Severe (<0.5 × 10^9^ neutrophils/L)	1 (<1)	1 (<1)	2 (<1)
**Myelosuppression intervention, n (%)**			
G-CSF ^§^	198 (50)	227 (53)	425 (52)
Erythropoietin ^¶^	28 (7)	37 (9)	65 (8)

* ECOG PS 5 excluded for deidentification purposes; ^†^ any time before index date; ^‡^ for patients with multiple neutropenia records on the same day, the minimum value was used; ^§^ included pegfilgrastim or filgrastim before index date; ^¶^ included epoetin alfa before index date. ECOG PS: Eastern Cooperative Oncology Group performance status, EGFR: epidermal growth factor receptor, G-CSF: granulocyte colony-stimulating factor, IQR: interquartile range; R: Regorafenib, T: TAS-102 ± bevacizumab.

**Table 2:** Neutropenia and myelosuppression medical interventions by cohort.

	R-T n = 393	T-R n = 425	Overall n = 818
Neutropenia, n (%) *			
Normal (>1.5 × 10^9^/L)	146 (37)	123 (29)	269 (33)
Mild (1.0–1.5 × 10^9^/L)	3 (1)	1 (<1)	4 (<1)
Moderate (0.5–0.9 × 10^9^/L)	102 (26)	135 (32)	237 (29)
Severe (<0.5 × 10^9^/L)	48 (12)	68 (16)	116 (14)
Unknown/missing	94 (24)	98 (23)	192 (23)
**Myelosuppression intervention**			
G-CSF or erythropoietin taken during sequential	87 (22)	102 (24)	189 (23)
therapy period, n (%)			
Overall use of G-CSF, ^†^ n (%)	55 (14)	76 (18)	131 (16)
Incidence rate (per 1000 person-months) ^‡^	14.9	22.2	18.4

* Lowest neutrophil count (neutrophils/L) measured during the sequential therapy period; ^†^ G-CSF initiated from Day 0 to the end of the sequential therapy period; ^‡^ incidence rate was defined as the number of new cases of G-CSF use during the sequential therapy period, regardless of previous use of G-CSF at baseline/total person-months of the corresponding cohort during the sequential therapy period. G-CSF: granulocyte colony-stimulating factor, R: Regorafenib, T: TAS-102 ± bevacizumab.

**Table 3:** Subsequent therapies.

	R-T n = 393	T-R n = 425	Overall n = 818
**Patients receiving subsequent therapy or therapies, n (%)**	133 (34)	149 (35)	282 (34)
Type of subsequent therapies, n (%) *	n = 133	n = 149	n = 282
Chemotherapy ^†^	42 (32)	59 (40)	101 (36)
Chemotherapy ^†^ + bevacizumab	34 (26)	53 (36)	87 (31)
Chemotherapy + targeted therapy ^‡^	27 (20)	34 (23)	61 (22)
Targeted therapy ^‡^	32 (24)	15 (10)	47 (17)
IO ^§^	22 (17)	24 (16)	46 (16)
IO ^§^ + chemotherapy ^†^ or IO ^§^ + targeted therapy ^‡^	5 (4)	6 (4)	11 (4)
Triplet combinations of the above	5 (4)	1 (1)	6 (2)
Other ^¶^	2 (2)	3 (2)	5 (2)
**Number of subsequent lines of therapy, n (%)**	n = 133	n = 149	n = 282
1	96 (72)	102 (68)	198 (70)
2	23 (17)	32 (21)	55 (20)
≥3	14 (11)	15 (10)	29 (10)
Median (IQR)	1 (1, 2)	1 (1, 2)	1 (1, 2)
Range	1–5	1–5	1–5

* Patients with >1 subsequent line of therapy may be counted multiple times based on the subsequent therapy type in each line of therapy; ^†^ chemotherapies included capecitabine, CAPEOX, docetaxel, fluorouracil, FOLFIRI, FOLFOX, FOLFOXIRI, gemcitabine, irinotecan, leucovorin (folinic acid), mitomycin, paclitaxel, sotorasib, and Trifluridine/Tipiracil; ^‡^ targeted therapies (not including bevacizumab) included afatinib, binimetinib, cabozantinib, cetuximab, encorafenib, lapatinib, lenvatinib, olaparib, panitumumab, pazopanib, pertuzumab, ramucirumab, Regorafenib, rituximab, sunitinib, trametinib, trastuzumab, vemurafenib, and ziv-aflibercept; ^§^ immunotherapies included ipilimumab, nivolumab, and pembrolizumab; ^¶^ other therapies included anastrozole, bicalutamide, letrozole, and leuprolide. CAPEOX: oxaliplatin and capecitabine, FOLFIRI: leucovorin, fluorouracil, and irinotecan, FOLFOX: leucovorin, fluorouracil, and oxaliplatin, FOLFOXIRI: leucovorin, fluorouracil, oxaliplatin, and irinotecan, IO: immunotherapy, IQR: interquartile range, R: Regorafenib, T: TAS-102 ± bevacizumab.

## Data Availability

The data that support the findings of this study were originated by and are the property of Flatiron Health, Inc. (New York, NY, USA), which has restrictions prohibiting the authors from making the data set publicly available. Requests for data sharing by license or by permission for the specific purpose of replicating results in this manuscript can be submitted to PublicationsDataAccess@flatiron.com.

## Supplementary Materials

The following supporting information can be downloaded at: https://www.mdpi.com/article/10.3390/cancers17060969/s1, Table S1: Patient characteristics at baseline by age; Table S2: Patient characteristics at baseline in patients receiving third- and fourth-line treatment; Table S3: Subgroup analysis of OS among third- and fourth-line patients; Table S4: Subgroup analysis of TTD among third- and fourth-line patients; Figure S1: Patient attrition.

## References

[1] National Cancer Institute Cancer Stat Facts: Colorectal Cancer. https://seer.cancer.gov/statfacts/html/colorect.html.

[2] Biller L.H., Schrag D. (2021). Diagnosis and treatment of metastatic colorectal cancer: A review. JAMA.

[3] Riedesser J.E., Ebert M.P., Betge J. (2022). Precision medicine for metastatic colorectal cancer in clinical practice. Ther. Adv. Med. Oncol..

[4] Grothey A. (2019). Insights into the mechanism of action of Regorafenib in colorectal cancer. Clin. Adv. Hematol. Oncol..

[5] Grothey A., Van Cutsem E., Sobrero A., Siena S., Falcone A., Ychou M., Humblet Y., Bouche O., Mineur L., Barone C. (2013). Regorafenib monotherapy for previously treated metastatic colorectal cancer (CORRECT): An international, multicentre, randomised, placebo-controlled, phase 3 trial. Lancet.

[6] Mayer R.J., Van Cutsem E., Falcone A., Yoshino T., Garcia-Carbonero R., Mizunuma N., Yamazaki K., Shimada Y., Tabernero J., Komatsu Y. (2015). Randomized trial of TAS-102 for refractory metastatic colorectal cancer. N. Engl. J. Med..

[7] Sastre J., Argiles G., Benavides M., Feliu J., Garcia-Alfonso P., Garcia-Carbonero R., Gravalos C., Guillen-Ponce C., Martinez-Villacampa M., Pericay C. (2014). Clinical management of Regorafenib in the treatment of patients with advanced colorectal cancer. Clin. Transl. Oncol..

[8] U.S. Food and Drug Administration Stivarga (Regorafenib) US Prescribing Information 2020. https://www.accessdata.fda.gov/drugsatfda_docs/label/2020/203085s011lbl.pdf.

[9] U.S. Food and Drug Administration Lonsurf (Trifluridine/Tipiracil) US Prescribing Information 2019. https://www.accessdata.fda.gov/drugsatfda_docs/label/2019/207981s008lbl.pdf.

[10] European Medicines Agency Lonsurf (Trifluridine/Tipiracil) Summary of Product Characteristics 2023. https://www.ema.europa.eu/en/documents/product-information/lonsurf-epar-product-information_en.pdf.

[11] European Medicines Agency Stivarga (Regorafenib) Summary of Product Characteristics 2023. https://www.ema.europa.eu/en/documents/product-information/stivarga-epar-product-information_en.pdf.

[12] Tabernero J., Prager G.W., Fakih M., Ciardiello F., Van Cutsem E., Elez E., Cruz M., Wyrwicz L., Stroyakosvskiy D., Papal Z. (2023). Trifluridine/Tipiracil plus bevacizumab for third-line treatment of refractory metastatic colorectal cancer. The phase 3 randomized SUNLIGHT study. J. Clin. Oncol..

[13] Bekaii-Saab T., Kim R., Kim T.W., O’Connor J.M., Strickler J.H., Malka D., Sartore-Bianchi A., Bi F., Yamaguchi K., Yoshino T. (2019). Third- or later-line therapy for metastatic colorectal cancer: Reviewing best practice. Clin. Color. Cancer.

[14] Donnini S., Filippelli A., Ciccone V., Spini A., Ristori E., Ziche M., Morbidelli L., Morbidelli L. (2022). Chapter 2—Antiangiogenic drugs: Chemosensitizers for combination cancer therapy. Antiangiogenic Drugs as Chemosensitizers in Cancer Therapy.

[15] Signorelli C., Calegari M.A., Basso M., Anghelone A., Lucchetti J., Minelli A., Angotti L., Zurlo I.V., Schirripa M., Chilelli M.G. (2023). Treatment settings and outcomes with Regorafenib and Trifluridine/Tipiracil at third-line treatment and beyond in metastatic colorectal cancer: A real-world multicenter retrospective study. Curr. Oncol..

[16] Su G.L., Wang Y.Y., Wang J.C., Liu H. (2020). A meta-analysis comparing Regorafenib with TAS-102 for treating refractory metastatic colorectal cancer. J. Int. Med. Res..

[17] Oshima K., Hirano H., Shoji H., Iwasa S., Okita N., Takashima A., Boku N. (2022). Influence of precedent drug on the subsequent therapy in the sequence of Trifluridine/Tipiracil with/out bevacizumab and Regorafenib for unresectable or recurrent colorectal cancer. PLoS ONE.

[18] Unseld M., Drimmel M., Siebenhuner A., Gleiss A., Bianconi D., Kieler M., Scheithauer W., Winder T., Prager G.W. (2018). Optimizing treatment sequence for late-line metastatic colorectal cancer patients using Trifluridine/Tipiracil and Regorafenib. Clin. Color. Cancer.

[19] Nevala-Plagemann C., Sama S., Ying J., Shen J., Haaland B., Florou V., Garrido-Laguna I. (2023). A real-world comparison of Regorafenib and Trifluridine/Tipiracil in refractory metastatic colorectal cancer in the United States. J. Natl. Compr. Cancer Netw..

[20] Vitale P., Zanaletti N., Famiglietti V., De Falco V., Cervantes A., Rosello S., Fenocchio E., Milanesio M., Lombardi P., Ciardiello D. (2021). Retrospective Study of Regorafenib Versus TAS-102 Efficacy and Safety in Chemorefractory Metastatic Colorectal Cancer (mCRC) Patients: A Multi-institution Real Life Clinical Data. Clin. Color. Cancer.

[21] Coutzac C., Trouilloud I., Artru P., Henriques J., Masson T., Doat S., Bouche O., Coriat R., Saint A., Moulin V. (2022). Sequential Treatment with Trifluridine/Tipiracil and Regorafenib in Refractory Metastatic Colorectal Cancer Patients: An AGEO Prospective “Real-World Study”. Clin. Color. Cancer.

[22] Ottaiano A., Santorsola M., Perri F., Granata V., Cascella M., Sabbatino F., Nasti G. (2023). Survival and toxicities of metastatic colorectal cancer patients treated with Regorafenib before TAS-102 or vice versa: A mono-institutional real-practice study. J. Clin. Med..

[23] Chida K., Kotani D., Moriwaki T., Fukuoka S., Masuishi T., Takashima A., Kumekawa Y., Kajiwara T., Yamazaki K., Komoda M. (2021). Survival benefit of crossover administration of Regorafenib and Trifluridine/Tipiracil hydrochloride for patients with metastatic colorectal cancer: Exploratory analysis of a Japanese Society for Cancer of the Colon and Rectum Multicenter Observational Study (REGOTAS). Front. Oncol..

[24] Ma X., Long L., Moon S., Adamson B.J.S., Baxi S.S. (2023). Comparison of population characteristics in real-world clinical oncology databases in the US: Flatiron Health, SEER, and NPCR. medRxiv.

[25] Birnbaum B., Nussbaum N., Seidl-Rathkopf K., Agrawal M., Estevez M., Estola E., Haimson J., He L., Larson P., Richardson P. (2020). Model-assisted cohort selection with bias analysis for generating large-scale cohorts from the EHR for oncology research. arXiv.

[26] Li J., Qin S., Xu R., Yau T.C., Ma B., Pan H., Xu J., Bai Y., Chi Y., Wang L. (2015). Regorafenib plus best supportive care versus placebo plus best supportive care in Asian patients with previously treated metastatic colorectal cancer (CONCUR): A randomised, double-blind, placebo-controlled, phase 3 trial. Lancet Oncol..

[27] Abrahao A.B.K., Ko Y.J., Berry S., Chan K.K.W. (2018). A comparison of Regorafenib and TAS-102 for metastatic colorectal cancer: A systematic review and network meta-analysis. Clin. Colorectal Cancer.

[28] Zeineddine F.A., Zeineddine M.A., Yousef A., Gu Y., Chowdhury S., Dasari A., Huey R.W., Johnson B., Kee B., Lee M.S. (2023). Survival improvement for patients with metastatic colorectal cancer over twenty years. NPJ Precis. Oncol..

[29] Dioca M., O’Connor J.M. (2021). Optimizing Regorafenib dosing and patient management in colorectal cancer in Latin America: Perspectives from Argentina. Oncologist.

[30] Kato T., Kudo T., Kagawa Y., Murata K., Ota H., Noura S., Hasegawa J., Tamagawa H., Ohta K., Ikenaga M. (2023). Phase II dose titration study of Regorafenib in progressive unresectable metastatic colorectal cancer. Sci. Rep..

[31] Argiles G., Mulet N., Valladares-Ayerbes M., Vieitez J.M., Gravalos C., Garcia-Alfonso P., Santos C., Tobena M., Garcia-Paredes B., Benavides M. (2022). A randomised phase 2 study comparing different dose approaches of induction treatment of Regorafenib in previously treated metastatic colorectal cancer patients (REARRANGE trial). Eur. J. Cancer.

[32] Bekaii-Saab T.S., Ou F.S., Ahn D.H., Boland P.M., Ciombor K.K., Heying E.N., Dockter T.J., Jacobs N.L., Pasche B.C., Cleary J.M. (2019). Regorafenib dose-optimisation in patients with refractory metastatic colorectal cancer (ReDOS): A randomised, multicentre, open-label, phase 2 study. Lancet Oncol..

