# Supplementary material for: Sequential Treatment with Regorafenib and Trifluridine/Tipiracil ± Bevacizumab in Refractory Metastatic Colorectal Cancer in Community Clinical Practice in the USA
Source: Cancers (Basel). 2025 Mar 13;17(6):969. doi: 10.3390/cancers17060969 (PMC11939964; doi:10.3390/cancers17060969)
Supplement: Supplementary file 1 [file cancers-17-00969-s001.zip › cancers-3417288-supplementary.pdf]

Supplementary Material

Supplementary Table S1. Patient characteristics at baseline by age.

Supplementary Table S2. Patient characteristics at baseline in patients receiving third- and fourth-line treatment.

Supplementary Table S3. Subgroup analysis of OS among third- and fourth-line patients.

Supplementary Table S4. Subgroup analysis of TTD among third- and fourth-line patients.

Supplementary Figure S1. Patient attrition.

Supplementary Table S1. Patient characteristics at baseline by age.

	Age ≤65			Age >65		
	**R-T** n = 225	**T-R** n = 246	**Overall** N = 471	**R-T** n = 168	**T-R** n = 179	**Overall** N = 347
**Median age (IQR), years**	57 (52, 61)	57 (52, 61)	57 (52, 61)	71 (69, 75)	74 (70, 78)	73 (69, 77)
**Gender, n (%)**						
Male	133 (59)	127 (52)	260 (55)	100 (60)	100 (56)	200 (58)
Female	92 (41)	119 (48)	211 (45)	68 (40)	79 (44)	147 (42)
**Race, n (%)**						
White	134 (60)	158 (64)	292 (62)	107 (64)	112 (63)	219 (63)
Black or African American	38 (17)	36 (15)	74 (16)	26 (15)	18 (10)	44 (13)
Asian	8 (4)	7 (3)	15 (3)	11 (7)	9 (5)	20 (6)
Other	25 (11)	28 (11)	53 (11)	19 (11)	27 (15)	46 (13)
Unknown/missing	20 (9)	17 (7)	37 (8)	5 (3)	13 (7)	18 (5)
**Side of primary tumor, n (%)**						
Left	150 (67)	145 (59)	295 (62)	90 (54)	96 (54)	186 (54)
Right	58 (26)	53 (22)	111 (24)	54 (32)	62 (35)	116 (33)
Unknown/missing	17 (8)	48 (20)	65 (14)	24 (14)	21 (12)	45 (13)
**Site of metastasis, n (%)**						
Liver ± other metastatic sites	162 (72)	161 (65)	323 (69)	108 (64)	121 (68)	229 (66)

Non-liver only	53 (24)	50 (20)	103 (22)	41 (24)	41 (23)	82 (24)
Unknown/missing	10 (4)	35 (14)	45 (10)	19 (11)	17 (10)	36 (10)
**Stage at initial diagnosis, n (%)**						
0/I	7 (3)	8 (3)	15 (3)	6 (4)	4 (2)	10 (3)
II	20 (9)	20 (8)	40 (8)	19 (11)	30 (17)	49 (14)
III	61 (27)	59 (24)	120 (25)	55 (33)	60 (34)	115 (33)
IV	134 (60)	155 (63)	289 (61)	83 (49)	82 (46)	165 (48)
Unknown/missing	3 (1)	4 (2)	7 (1)	5 (3)	3 (2)	8 (2)
**ECOG PS, n (%)**						
0/1	155 (69)	180 (73)	335 (71)	123 (73)	120 (67)	243 (70)
2/3	17 (8)	19 (8)	36 (8)	14 (8)	24 (13)	38 (11)
Unknown/missing*	53 (24)	47 (19)	100 (21)	31 (18)	35 (20)	66 (19)
***KRAS* mutation, n (%)†**	109 (48)	110 (45)	219 (46)	74 (44)	84 (47)	158 (46)
***BRAF* mutation, n (%)†**	2 (1)	12 (5)	14 (3)	3 (2)	7 (4)	10 (3)
**Line of index treatment, n (%)**						
1	14 (6)	14 (6)	28 (6)	12 (7)	13 (7)	25 (7)
2	38 (17)	31 (13)	69 (15)	29 (17)	36 (20)	65 (19)
3	93 (41)	106 (43)	199 (42)	74 (44)	70 (39)	144 (41)
4	51 (23)	68 (28)	119 (25)	36 (21)	39 (22)	75 (22)
5–8	29 (13)	27 (11)	56 (12)	17 (10)	21 (12)	38 (11)

**Time between mCRC diagnosis and index date, median months (IQR)**	26.4 (16.7, 36.5)	25.3 (17.7, 35.9)	25.6 (17.2, 36.0)	23.2 (16.5, 34.4)	24.2 (16.8, 37.0)	23.8 (16.8, 35.7)
**Prior anti-EGFR, n (%)**	74 (33)	99 (40)	173 (37)	57 (34)	54 (30)	111 (32)
**Prior bevacizumab, n (%)**	176 (78)	200 (81)	376 (80)	132 (79)	137 (77)	269 (78)
**Neutropenia, n (%)[‡]**						
Moderate (0.5–1 x 10^9 neutrophils/L)	8 (4)	4 (2)	12 (3)	6 (4)	4 (2)	10 (3)
Severe (<0.5 x 10^9 neutrophils/L)	0	1 (<1)	1 (<1)	1 (1)	0	1 (<1)
**Myelosuppression intervention, n (%)**						
G-CSF[§]	113 (50)	126 (51)	239 (51)	85 (51)	101 (56)	186 (54)
Erythropoietin[¶]	13 (6)	16 (7)	29 (6)	15 (9)	21 (12)	36 (10)

[*]ECOG PS 5 excluded for deidentification purposes; [†]Any time before index date; [‡]For patients with multiple neutropenia records on the same day, the minimum value was used; [§]Included pegfilgrastim or filgrastim before index date; [¶]Included epoetin alfa before index date.

ECOG PS: Eastern Cooperative Oncology Group performance status, EGFR: epidermal growth factor receptor, G-CSF: granulocyte colony-stimulating factor, IQR: interquartile range, mCRC: metastatic colorectal cancer, R: regorafenib, T: TAS-102 ± bevacizumab.

Supplementary Table S2. Patient characteristics at baseline in patients receiving third- and fourth-line treatment.

	Third line			Fourth line		
	**R-T n = 167**	**T-R n = 176**	**Overall N = 343**	**R-T n = 87**	**T-R n = 107**	**Overall N = 194**
**Median age (IQR), years**	62 (55, 71)	63 (56, 70)	63 (56, 71)	63 (58, 69)	62 (55, 71)	63 (56, 70)
**Gender, n (%)**						
Male	96 (57)	87 (49)	183 (53)	60 (69)	62 (58)	122 (63)
Female	71 (43)	89 (51)	160 (47)	27 (31)	45 (42)	72 (37)
**Race, n (%)**						
White	106 (63)	107 (61)	213 (62)	46 (53)	71 (66)	117 (60)
Black or African American	29 (17)	24 (14)	53 (15)	18 (21)	14 (13)	32 (16)
Asian	8 (5)	6 (3)	14 (4)	6 (7)	5 (5)	11 (6)
Other	13 (8)	28 (16)	41 (12)	12 (14)	9 (8)	21 (11)
Unknown/missing	11 (7)	11 (6)	22 (6)	5 (6)	8 (7)	13 (7)
**Side of primary tumor, n (%)**						
Left	100 (60)	95 (54)	195 (57)	58 (67)	63 (59)	121 (62)
Right	50 (30)	60 (34)	110 (32)	18 (21)	20 (19)	38 (20)
Unknown/missing	17 (10)	21 (12)	38 (11)	11 (13)	24 (22)	35 (18)
**Site of metastasis, n (%)**						
Liver ± other metastatic sites	121 (72)	124 (70)	245 (71)	60 (69)	66 (62)	126 (65)

Non-liver only	34 (20)	41 (23)	75 (22)	18 (21)	20 (19)	38 (20)
Unknown/missing	12 (7)	11 (6)	23 (7)	9 (10)	21 (20)	30 (15)
**Stage at initial diagnosis, n (%)**						
0/I	5 (3)	4 (2)	9 (3)	4 (5)	4 (4)	8 (4)
II	18 (11)	21 (12)	39 (11)	7 (8)	12 (11)	19 (10)
III	44 (26)	51 (29)	95 (28)	18 (21)	17 (16)	35 (18)
IV	96 (57)	96 (55)	192 (56)	58 (67)	74 (69)	132 (68)
Unknown/missing	4 (2)	4 (2)	8 (2)	0	0	0
**ECOG PS, n (%)**						
0/1	132 (79)	128 (73)	260 (76)	64 (74)	73 (68)	137 (71)
2/3	10 (6)	18 (10)	28 (8)	7 (8)	15 (14)	22 (11)
Unknown/missing*	25 (15)	30 (17)	55 (16)	16 (18)	19 (18)	35 (18)
***KRAS* mutation, n (%)**	87 (52)	97 (55)	184 (54)	34 (39)	35 (33)	69 (36)
***BRAF* mutation, n (%)**	2 (1)	12 (7)	14 (4)	2 (2)	6 (6)	8 (4)
**Time between mCRC diagnosis and index date, median months (IQR)**	23.3 (15.4, 33.6)	21.7 (15.1, 32.8)	22.4 (15.1, 33.3)	29.8 (21.5, 40.5)	28.5 (20.7, 38.8)	29.2 (21.0, 38.9)
**Prior anti-EGFR, n (%)†**	52 (31)	52 (30)	104 (30)	43 (49)	63 (59)	106 (55)
**Prior bevacizumab, n (%)†**	147 (88)	154 (88)	301 (88)	79 (91)	95 (89)	174 (90)

**Neutropenia, n (%)**[‡]						
Moderate (0.5–1 x 10^9 neutrophils/L)	6 (4)	3 (2)	9 (3)	6 (7)	2 (2)	8 (4)
Severe (<0.5 x 10^9 neutrophils/L)	0	0	0	0	0	0
**Myelosuppression intervention, n (%)**						
G-CSF[§]	97 (58)	96 (55)	193 (56)	46 (53)	66 (62)	112 (58)
Erythropoietin[¶]	10 (6)	13 (7)	23 (7)	9 (10)	13 (12)	22 (11)

[*]ECOG PS 5 excluded for deidentification purposes; [†]Any time before index date; [‡]For patients with multiple neutropenia records on the same day, the minimum value was used; [§]Included pegfilgrastim or filgrastim before index date; [¶]Included epoetin alfa before index date.

ECOG PS: Eastern Cooperative Oncology Group performance status, EGFR: epidermal growth factor receptor, G-CSF: granulocyte colony-stimulating factor, IQR: interquartile range, mCRC: metastatic colorectal cancer, R: regorafenib, T: TAS-102 ± bevacizumab.

Supplementary Table S3. Subgroup analysis of OS among third- and fourth-line patients.

		R-T	T-R
		n = 110	n = 109
**Age >65 years**	Follow-up time for OS (median), months	10.8	10.2
	(IQR)	(7.4, 19.7)	(6.8, 18.4)
	OS (median, KM method), months	12.3	11.3
	(95% CI)	(10.7–14.0)	(9.5–13.9)
	Unadjusted HR (R-T vs. T-R)*	0.92 (0.69, 1.24)	
	Adjusted HR (R-T vs. T-R)*†	1.00 (0.69, 1.46)	
		n = 144	n = 174
**Age ≤65 years**	Follow-up time for OS (median), months	11.0	9.8
	(IQR)	(7.3, 17.0)	(6.5, 15.4)
	OS (median, KM method), months	12.3	11.3
	(95% CI)	(10.5–13.7)	(9.9–13.2)
	Unadjusted HR (R-T vs. T-R)*	0.88 (0.69, 1.13)	
	Adjusted HR (R-T vs. T-R)*†	0.97 (0.72, 1.31)	
**Third-line**		n = 167	n = 176
**index therapy**	Follow-up time for OS (median), months	11.2	10.1
	(IQR)	(6.8, 18.6)	(6.5, 16.5)
	OS (median, KM method), months	13.1	11.5
	(95% CI)	(11.0–15.1)	(10.4–13.7)

	Unadjusted HR (R-T vs. T-R)*	0.94 (0.74, 1.19)	
	Adjusted HR (R-T vs. T-R)*†	1.02 (0.76, 1.36)	
**Fourth-line index therapy**		n = 87	n = 107
	Follow-up time for OS (median), months	10.4	9.8
	(IQR)	(7.4, 14.9)	(6.9, 14.8)
	OS (median, KM method), months	11.6	10.3
	(95% CI)	(9.6–13.2)	(8.9–12.6)
	Unadjusted HR (R-T vs. T-R)*	0.86 (0.63, 1.18)	
	Adjusted HR (R-T vs. T-R)*†	0.99 (0.65, 1.50)	
**Prior bevacizumab**		n = 226	n = 249
	Follow-up time for OS (median), months	11.1	9.8
	(IQR)	(7.3, 17.1)	(6.6, 15.4)
	OS (median, KM method), months	12.3	10.9
	(95% CI)	(11.0–13.4)	(9.8–13.1)
	Unadjusted HR (R-T vs. T-R)*	0.88 (0.72, 1.08)	
	Adjusted HR (R-T vs. T-R)*†	1.02 (0.80, 1.30)	
**No prior bevacizumab**		n = 28	n = 34
	Follow-up time for OS (median), months	9.8	11.9
	(IQR)	(8.8, 16.0)	(8.4, 19.5)
	OS (median, KM method), months	11.4	13.1
	(95% CI)	(9.3–19.4)	(10.0–16.5)

	Unadjusted HR (R-T vs. T-R)*	0.97 (0.53, 1.79)	
	Adjusted HR (R-T vs. T-R)*†	0.78 (0.26, 2.31)	
		n = 116	n = 130
***KRAS* wild type**	Follow-up time for OS (median), months (IQR)	10.9 (7.6, 17.0)	10.4 (7.3, 15.7)
	OS (median, KM method), months (95% CI)	12.1 (10.6–13.7)	12.9 (10.5–15.4)
	Unadjusted HR (R-T vs. T-R)*	0.86 (0.65, 1.15)	
	Adjusted HR (R-T vs. T-R)*†	0.97 (0.68, 1.37)	
		n = 121	n = 132
***KRAS* mutant**	Follow-up time for OS (median), months (IQR)	10.5 (7.0, 15.9)	9.2 (6.2, 16.4)
	OS (median, KM method), months (95% CI)	11.6 (10.3–13.5)	9.9 (8.6–13.1)
	Unadjusted HR (R-T vs. T-R)*	1.01 (0.77, 1.33)	
	Adjusted HR (R-T vs. T-R)*†	1.04 (0.75, 1.45)	
		n = 181	n = 190
**Liver metastasis**	Follow-up time for OS (median), months (IQR)	9.6 (6.8, 15.1)	8.8 (6.4, 14.2)

**(with or without other metastatic sites)**	OS (median, KM method), months<br>(95% CI)	11.2<br>(9.5–12.9)	10.4<br>(8.8–11.7)
	Unadjusted HR (R-T vs. T-R)*	0.88 (0.70, 1.11)	
	Adjusted HR (R-T vs. T-R)*†	0.95 (0.72, 1.24)	
**Non-liver metastasis only**		n = 52	n = 61
	Follow-up time for OS (median), months<br>(IQR)	13.4<br>(9.6, 21.1)	11.8<br>(8.1, 20.5)
	OS (median, KM method), months<br>(95% CI)	14.1<br>(12.3–18.7)	14.7<br>(10.1–18.4)
	Unadjusted HR (R-T vs. T-R)*	0.90 (0.59, 1.36)	
	Adjusted HR (R-T vs. T-R)*†	1.10 (0.59, 2.03)	
**Left-sided tumor**		n = 158	n = 158
	Follow-up time for OS (median), months<br>(IQR)	11.0<br>(7.4, 17.1)	10.0<br>(6.8, 15.2)
	OS (median, KM method), months<br>(95% CI)	12.4<br>(10.7–13.3)	11.4<br>(9.8–13.1)
	Unadjusted HR (R-T vs. T-R)*	0.92 (0.73, 1.18)	
	Adjusted HR (R-T vs. T-R)*†	1.06 (0.79, 1.42)	
**Right-sided tumor**		n = 68	n = 80
	Follow-up time for OS (median), months	9.5	8.6

(IQR)	(6.3, 16.4)	(5.8, 17.1)
OS (median, KM method), months	11.3	10.8
(95% CI)	(9.2–15.1)	(8.2–14.8)
Unadjusted HR (R-T vs. T-R)*	0.90 (0.62, 1.30)	
Adjusted HR (R-T vs. T-R)*†	0.96 (0.63, 1.49)	

*Supremum test for proportional hazards assumption *p*-value >0.05, proportional hazards assumption not violated; †Adjusted for index line, age, gender, ECOG PS, *KRAS* mutation status, prior anti-EGFR, prior bevacizumab, stage at initial diagnosis, tumor sidedness, and site of metastasis. The corresponding covariate corresponding to the subgroup is removed from the model.

CI: confidence interval, ECOG PS: Eastern Cooperative Oncology Group performance status, EGFR: epidermal growth factor receptor, HR: hazard ratio, IQR: interquartile range, KM: Kaplan-Meier, OS: overall survival, R: regorafenib, T: TAS-102 ± bevacizumab.

Supplementary Table S4. Subgroup analysis of TTD among third- and fourth-line patients.

		R-T	T-R
		n = 110	n = 109
**Age >65 years**	Follow-up time for TTD (median), months	8.2	7.4
	(IQR)	(6.0, 12.8)	(5.8, 10.0)
	TTD (median, KM method), months	8.7	7.7
	(95% CI)	(7.5–10.4)	(6.9–9.1)
	Unadjusted HR (R-T vs. T-R)*	0.91 (0.68, 1.20)	
	Adjusted HR (R-T vs. T-R)*†	1.03 (0.72, 1.46)	
		n = 144	n = 174
**Age ≤65 years**	Follow-up time for TTD (median), months	8.3	7.5
	(IQR)	(5.2, 11.4)	(5.2, 10.5)
	TTD (median, KM method), months	8.4	8.1
	(95% CI)	(7.8–9.5)	(7.3–9.0)
	Unadjusted HR (R-T vs. T-R)*	0.91 (0.72, 1.15)	
	Adjusted HR (R-T vs. T-R)*†	1.01 (0.75, 1.36)	
**Third-line**		n = 167	n = 176
**index therapy**	Follow-up time for TTD (median), months	8.3	7.2
	(IQR)	(5.6, 12.2)	(5.2, 10.3)
	TTD (median, KM method), months	8.7	8.1
	(95% CI)	(7.8–9.8)	(6.9–9.0)

	Unadjusted HR (R-T vs. T-R)*	0.91 (0.73, 1.14)	
	Adjusted HR (R-T vs. T-R)*†	1.01 (0.83, 1.45)	
**Fourth-line index therapy**		n = 87	n = 107
	Follow-up time for TTD (median), months (IQR)	8.2 (6.0, 11.7)	7.6 (6.0, 10.0)
	TTD (median, KM method), months (95% CI)	8.5 (7.3–10.0)	7.9 (7.3–9.1)
	Unadjusted HR (R-T vs. T-R)*	0.86 (0.63, 1.16)	
	Adjusted HR (R-T vs. T-R)*†	0.85 (0.56, 1.27)	
**Prior bevacizumab**		n = 226	n = 249
	Follow-up time for TTD (median), months (IQR)	8.2 (5.8, 12.2)	7.2 (5.3, 10.0)
	TTD (median, KM method), months (95% CI)	8.5 (7.7–9.5)	7.7 (7.1–8.7)
	Unadjusted HR (R-T vs. T-R)*	0.88 (0.73, 1.07)	
	Adjusted HR (R-T vs. T-R)*†	1.04 (0.82, 1.32)	
**No prior bevacizumab**		n = 28	n = 34
	Follow-up time for TTD (median), months (IQR)	8.6 (5.7, 11.0)	8.7 (6.7, 11.6)
	TTD (median, KM method), months (95% CI)	8.7 (6.0–11.1)	9.1 (7.6–9.9)

	Unadjusted HR (R-T vs. T-R)*	1.02 (0.60, 1.75)	
	Adjusted HR (R-T vs. T-R)*†	1.03 (0.39, 2.74)	
***KRAS* wild type**		n = 116	n = 130
	Follow-up time for TTD (median), months (IQR)	8.3 (6.0, 11.7)	7.8 (6.1, 10.2)
	TTD (median, KM method), months (95% CI)	8.5 (8.0–10.0)	8.8 (7.7–9.4)
	Unadjusted HR (R-T vs. T-R)*	0.95 (0.72, 1.24)	
	Adjusted HR (R-T vs. T-R)*†	1.11 (0.80, 1.53)	
***KRAS* mutant**		n = 121	n = 132
	Follow-up time for TTD (median), months (IQR)	7.6 (5.3, 11.5)	6.8 (4.8, 10.3)
	TTD (median, KM method), months (95% CI)	7.9 (6.9–9.4)	7.4 (6.4–8.5)
	Unadjusted HR (R-T vs. T-R)*	0.94 (0.73, 1.22)	
	Adjusted HR (R-T vs. T-R)*†	0.99 (0.73, 1.36)	
**Liver metastasis (with or without other**		n = 181	n = 190
	Follow-up time for TTD (median), months (IQR)	7.5 (5.2, 10.6)	6.7 (5.1, 9.7)
	TTD (median, KM method), months (95% CI)	7.7 (7.2–8.4)	7.3 (6.6–7.9)

**metastatic sites)**	Unadjusted HR (R-T vs. T-R)*	0.89 (0.72, 1.10)	
	Adjusted HR (R-T vs. T-R)*†	0.98 (0.76, 1.27)	
**Non-liver metastasis only**		n = 52	n = 61
	Follow-up time for TTD (median), months (IQR)	10.4 (7.6, 16.8)	9.1 (6.4, 11.9)
	TTD (median, KM method), months (95% CI)	11.7 (9.4–15.3)	9.2 (8.0–10.0)
	Unadjusted HR (R-T vs. T-R)*	0.85 (0.57, 1.27)	
	Adjusted HR (R-T vs. T-R)*†	1.19 (0.67, 2.09)	
**Left-sided tumor**		n = 158	n = 158
	Follow-up time for TTD (median), months (IQR)	8.3 (5.8, 12.0)	7.4 (5.7, 9.8)
	TTD (median, KM method), months (95% CI)	8.4 (7.8–9.6)	7.7 (7.2–8.8)
	Unadjusted HR (R-T vs. T-R)*	0.79 (0.62, 1.00)	
	Adjusted HR (R-T vs. T-R)*†	1.01 (0.76, 1.35)	
**Right-sided tumor**		n = 68	n = 80
	Follow-up time for TTD (median), months (IQR)	7.3 (5.0, 9.9)	6.6 (4.7, 11.0)
	TTD (median, KM method), months (95% CI)	7.6 (6.3–9.5)	7.6 (6.2–9.4)

	Unadjusted HR (R-T vs. T-R)*	1.09 (0.77, 1.55)
	Adjusted HR (R-T vs. T-R)*†	1.26 (0.83, 1.90)

*Supremum test for proportional hazards assumption *p*-value >0.05, proportional hazards assumption not violated; †Adjusted for index line, age, gender, ECOG PS, *KRAS* mutation status, prior anti-EGFR, prior bevacizumab, stage at initial diagnosis, tumor sidedness, and site of metastasis. The corresponding covariate corresponding to the subgroup is removed from the model.

CI: confidence interval, ECOG PS: Eastern Cooperative Oncology Group performance status, EGFR: epidermal growth factor receptor, HR: hazard ratio, IQR: interquartile range, KM: Kaplan-Meier, R: regorafenib, T: TAS-102 ± bevacizumab, TTD: time to treatment discontinuation.

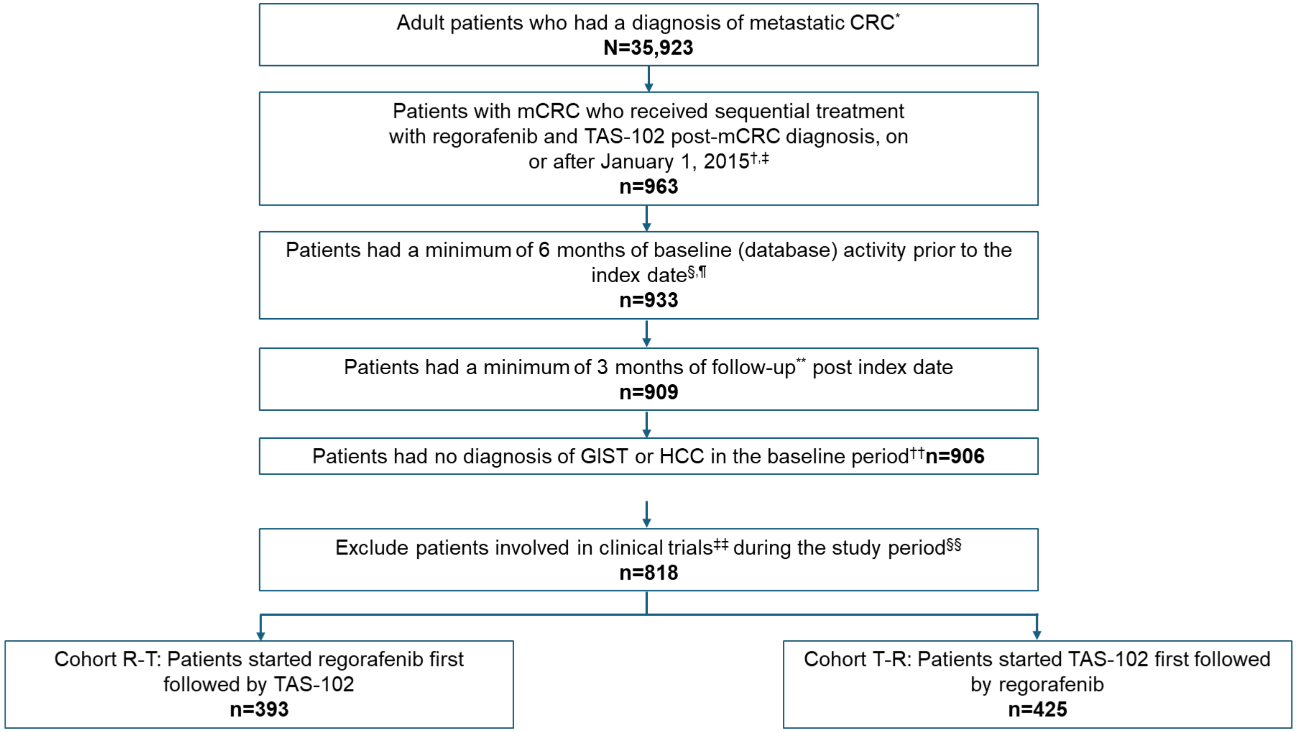

Supplementary Figure S1. Patient attrition.

*Data cut-off date February 28, 2023; [†]Regorafenib and TAS-102 treatment can be monotherapy or combination therapy; [‡]Patients did not receive any other line of therapy between regorafenib and TAS-102; [§]Index date was defined as the start of the line of treatment that contained first regorafenib or TAS-102; [¶]Patient-level first activity date was defined as the earliest date of confirmed activity date, structured activity date, and confirmed structured activity date; [**]Follow-up until the earliest of the following events: date of death, last activity date, or study end date. Last activity date was defined as the latest date of confirmed activity; [††]Baseline period for GIST and HCC was defined as ≤5 years prior to the index date; [‡‡]Involvement in clinical trials was identified by "clinical study drug" in the Flatiron line of treatment table; [§§]Study period was defined from the first activity date to the latest data cut-off date.

CRC: colorectal cancer, GIST: gastrointestinal stromal tumor, HCC: hepatocellular carcinoma, mCRC: metastatic colorectal cancer, R: regorafenib, T: TAS-102, TAS-102: trifluridine/tipiracil.
